# Characterization, expression and application of a zearalenone degrading enzyme from *Neurospora crassa*

**DOI:** 10.1186/s13568-018-0723-z

**Published:** 2018-12-20

**Authors:** Ke Bi, Wen Zhang, Zhizhuang Xiao, Dawei Zhang

**Affiliations:** 1 Shandong Provincial Key Laboratory of Microbial Engineering, College of Biotechnology, Qilu University of Technology (Shandong Academy of Sciences), Jinan, 250353 China; 2 Qingdao Red Cherry Biotech Ltd, Qingdao, 266111 China

**Keywords:** Zearalenone degradation, *Pichia pastoris*, High-density fermentation, Animal feed, Maize

## Abstract

A gene named *zenc*, encoding a zearalenone lactonase from *Neurospora crassa*, was over-expressed in *Pichia pastoris*. The *zenc* gene is 888-bp in length, encoding a 295-residue polypeptide. Purified ZENC has maximal activity at pH 8.0 and 45 °C, and is highly stable at pH 6.0–8.0 for 1 h at 37 °C. The activity of the secreted enzyme in shaken-flask fermentation was 40.0 U/ml. A high-density fermentation of the ZENC-producing recombinant strain was performed in a 30-l fermenter and the maximal enzyme activity reached 290.6 U/ml. The K_m_, V_max_ and specific activity toward zearalenone are 38.63 μM, 23.8 μM/s/mg and 530.4 U/mg, respectively. ZENC can resist metal ions and inhibitors to some extent. We applied the enzyme into three different kinds of animal feed. On addition of ZENC (800 U) to distillers dried grains with solubles (DDGS), maize by-products and corn bran (25 g), the concentration of zearalenone was reduced by 70.9%, 88.9% and 94.7% respectively. All these properties of ZENC are promising for applications in the animal feed and food industries.

## Introduction

Zearalenone (ZEN) is a nonsteroidal estrogenic mycotoxin which was first isolated by Stob et al. in 1962 and its structure was determined by Urry (Perry et al. 1970; Urry et al. 1966). It was initially used as a growth promoter in animal feed (Wilson et al. 1972), but now it is considered to have adverse effects, mainly on the reproduction of animals (Kleinova et al. 2002). This mycotoxin is a secondary metabolite of *Fusarium* species including *F. graminearum*, *F. culmorum*, *F. equiseti*, *F. semitectum* and *F. crookwellense*, particularly *F. graminearum*. All these species are soil fungi and common in temperate regions (Bennett and Klich 2003). ZEN contamination mainly occurs in maize and its co-products, but it has also been detected in some other cereals, including barley, wheat and broomcorn (Hussein and Brasel 2001). Cereals are contaminated during the cultivation period due to the *Fusarium* in soil. Contamination can also be caused by improper storage of cereals.

The economic losses to animal production and cultivation associated with ZEN contamination are enormous. The contamination also causes a series of food security problems. Though ZEN has a low acute toxicity, its negative impact on the reproduction of livestock is observed over time when ZEN contaminated fodder is fed to animals. ZEN can bind to estrogen receptors because its lactone ring is similar to the aromatic ring of estradiol (Fink-Gremmels and Malekinejad 2007). The issues of animal reproduction associated with estrogenic effects, such as low survival ratio of embryos, infertility and decreased litter weight, are induced by ZEN intake (Minervini and Dell’Aquila 2008). Pigs are more sensitive to ZEN than other animals such as rodents and ruminants. Besides having an adverse effect on the reproductive system, ZEN is also genotoxic and immunotoxic, and may lead to increased occurrence of hepatocellular and breast cancer (Ahamed et al. 2001; Ghedira-Chekir et al. 1998; Shier et al. 2001).

Currently, there are three methods of reducing the amount of ZEN in cereals, which can be classified as physical, chemical and biological. Physical absorption is one of the most widely-used methods to tackle high concentrations of ZEN in feedstuffs. Chemical methods such as alkali and ozone treatments have been reported (Bennett et al. 1980; Karlovsky et al. 2016; Numanoglu et al. 2013). Biological treatment is a promising method of degradation of ZEN. Direct use of a microbial strain to degrade ZEN has been reported (Fu et al. 2016). It was demonstrated that *Aspergillus niger* FS10 was able to remove ZEN from medium and corn steep liquor (Sun et al. 2014). Three kinds of enzyme have been found to be able to degrade ZEN-laccase (EC 1.10.3.2), lactono hydrolase (E.C.3.1.1) and 2-Cys peroxiredoxin (EC 1.11.1.15) (Loi et al. 2017; Takahashi-Ando et al. 2002, 2005). Lactono hydrolase was one of the most studied ZEN degrading enzyme, such as ZHD101 (Takahashi-Ando et al. 2002; Yang et al. 2017), ZHD (Peng et al. 2014; Xiang et al. 2016). Here, we expressed a zearalenone lactonase gene from *Neurospora crassa* in *P. pastoris* to yield an enzyme that can degrade ZEN.

## Materials and methods

### Strains, plasmids and reagents

ZEN was purchased from ROMER Labs (Austria), and it was dissolved in acetonitrile as a standard stock solution (500 μg/ml). Water was purified by Master-R-UVF (HHitech, China). Acetonitrile was chromatographically pure grade (Merck, Germany). *P. pastoris* GS115 (Invitrogen, USA) was used as the host strain for protein expression. The cloning plasmid pPIC9K (Invitrogen, USA) was used for heterologous expression of zearalenone lactonase in *P. pastoris*. Routine chemicals were of analytical grade (Sangon Biotech, China). Basal salts medium, buffered glycerol-complex medium (BMGY) and Yeast Extract Peptone Dextrose Medium (YPD) were prepared according to the instruction of *Pichia* Expression Kit and *Pichia* Fermentation Process Guidelines (Invitrogen, USA).

### Cloning and expression of *zenc* gene in *P. pastoris*

The codons of the native *zenc* gene (GenBank ID: XM_958243.3) were optimized based on *P. pastoris* codon usage to obtain more effective protein expression. The optimized gene *zenc* (GenBank ID: MH780140) was synthesized with addition of *Eco*RI and *Not*I restriction sites at both ends by GenScript (Nanjing, China). The synthesized gene fragment was cloned into pPIC9K between the *Eco*RI and *Not*I restriction sites and fused in-frame with the α-factor signal peptide. The resulting plasmid was linearized by *Sal*I and then transformed into *P. pastoris* GS115 competent cells by electroporation. The recombined plasmid was identified by restriction enzyme digestion and the gene was confirmed by PCR. The restriction sites were *Eco*RI and *Not*I respectively. The specific primers were ZENC-FP (5′ TACCGGCATAAGAGAGAAGG 3′) and ZENC-RP (5′ TTAGTTTTCACCAAGAAATGGCA 3′). The transformation mixtures were spread onto minimal dextrose plates to screen for positive recombinant cells expressing ZENC.

### Expression and purification of ZENC

The recombinant *P. pastoris* strain was cultured in 250-ml shaken flasks containing 25 ml BMGY medium for 24 h (30 °C, 200 rpm). Then, 1% methanol (v/v) was added to the culture every 24 h to induce expression of the target protein. The strain was induced for 72 h.

For purification of protein, the cell culture supernatant was collected by centrifugation at 10,000 rpm at 4 °C for 15 min. Protein was concentrated by adding Ammonium sulfate with a final saturation of 80%. The protein precipitate was dissolved in 10 mM Tris–HCl buffer (pH 7.5) and the solution was desalinated by ultracentrifugation using 10 kDa filter units (Millipore, USA). The target protein was collected and applied to a HiTrap Q HP column (GE, USA), and eluted with a linear gradient from 0 to 1 M NaCl. The purified protein was loaded onto polyacrylamide gel for SDS-PAGE analyzing, and a BCA Protein Assay Kit (Solarbio, China) was used to determine the concentration of protein.

### High-performance liquid chromatography (HPLC) analysis

The chromatographic system consisted of a Shimadzu LC-20AT pump and a Shimadzu RF-20A fluorescence detector. A Shim-Pack GIST C18 column (4.6 mm × 150 mm, 5 μm) was used for chromatographic separation. The mobile phase containing acetonitrile–methanol–water 46:8:46 (v/v/v) was used at a flow rate of 0.8 ml/min. The column was kept at 30 °C and the injection volume was 20 μl. The excitation and emission wavelengths were 274 and 440 nm, respectively. The concentration of ZEN was determined based on retention times and peak areas compared to ZEN standards dissolved in acetonitrile. A standard curve was generated for ZEN and the linear relation between peak area and concentration was in very good correlation.

### Degradation activity assay of ZEN

The activity assay was performed at 45 °C and pH 8.0. The total reaction volume was 500 μl, which included 25 μl enzyme solution, 455 μl of 50 mM Tris–HCl buffer (pH 8.0) and 20 μl ZEN standard solution (500 μg/ml). The reaction mixture was incubated at 45 °C for 5 min and then 500 μl acetonitrile were added to terminate the reaction. The remaining ZEN concentration in the mixture was quantified by the Shimadzu Analytical HPLC system (“High-performance liquid chromatography (HPLC) analysis” section). One ZENC unit (U/ml) was defined as the amount of enzyme necessary to degrade 1 μg ZEN in per minute at 45 °C, pH 8.0. ZEN degradation rate (%) = [10 − remaining ZEN concentration (μg/ml)]/10 × 100%. Results represent the averages of three independent experiments.

### Characterization and kinetic analysis of enzyme

To determine the optimal pH of ZENC activity, incubation was performed in 50 mM Na2HPO4-citric acid buffer (pH 2.2–7.5), 50 mM Tris–HCl (pH 7.5–9.0) and 50 mM borate buffer (pH 10) at 37 °C for 5 min. The pH stability of ZENC was estimated by determining the residual activity in standard conditions (pH 8.0, 45 °C, 5 min) after preincubation of enzyme solution in buffers at pH 2.2–10 at 37 °C for 1 h without substrate. The activity of ZENC without incubation was considered 100%. The optimal reaction temperature was determined by performing the reaction at 20–60 °C in 50 mM Tris–HCl (pH 8.0) for 5 min. The reaction was terminated by adding 500 μl acetonitrile and reaction mixture (20 μl) was analyzed by HPLC. The thermostability of ZENC was explored by measuring the residual enzymatic activity after incubating the enzyme in 50 mM Tris–HCl (pH 8.0) at temperature ranging from 20 to 80 °C without substrate for different periods of time. The enzyme stability at 37 °C, pH 8.0 was tested particularly. The enzyme activity was measured after ZENC was stored at 37 °C, pH 8.0 for different time. The enzymes activity of 0 h incubation was set as 100% activity. The effects of different metal ions and chemicals on the activity of ZENC were measured by adding 5 mM various metal salts (NaCl, CaCl, MnSO_4_, MgSO_4_, CuSO_4_) and regents (SDS, EDTA) to assay system (“Degradation activity assay of ZEN” section). 5, 10, 15 mM ZnSO_4_ was added to assay system separately. The system without any additive was used as a control.

Kinetic analysis was performed in 50 mM Tris–HCl buffer (pH 8.0) at ZEN concentrations ranging from 2 to 220 mM at 45 °C for 3 min. Kinetic parameters K_m_ and V_max_ were determined using the Hill equation: υ = V_max_/[1 + (K_m_/C)n], where υ is the enzyme velocity, C is the substrate concentration, K_m_ is the substrate concentration at the half-maximum velocity, n is the Hill coefficient (n = 1), and V_max_ is the maximal velocity. The data were fitted to a nonlinear curve using Origin Pro 8 software (OriginLab, USA).

### Expression of ZENC in a 30-l fermenter

To scale up ZENC production, high cell-density fermentation was performed according to the *Pichia* fermentation process guidelines (Invitrogen). A colony of the ZENC-producing strain was inoculated into 100 ml YPD medium and incubated in rotary shaker of 200 rpm at 30 °C for 24 h as the first seed culture. The second seed culture was prepared by transferring the first seed culture into 900 ml YPD medium and grown for 10 h at 30 °C. The second seed was transferred into the 30-l fermenter containing 19 l BMGY medium with 10 ml/l PTM1 solution. The system was maintained 30 °C, pH 6.0, with ammonia. The carbon source was exhausted approximately after 18 h and DO then rapidly increased to 70%, 12 ml/l PTM1 solution with 50% glycerol (w/v) was fed into the fermenter; the DO level was maintained at 20–30% by controlling the feeding rate. After the wet weight of cells reached 200 g/l, pure methanol feeding (instead of glycerol feeding) commenced to induce expression of the target protein. Cell culture was collected every 12 h to determine the ZENC activity.

### Application of ZENC on degradation of ZEN in distillers dried grains with solubles (DDGS), maize by-products and corn bran

DDGS, maize by-products and corn bran (25 g) were respectively mixed with 20 ml crude enzyme (40.0 U/ml, pH 6.2) and adjusted to pH 8.0 by adding NaOH dissolved in 5 ml water. In the control, sample (25 g) was mixed with 20 ml inactive crude enzyme (inactivated by boiling-heat for 10 min, pH 6.2) and adjusted to pH 8.0 by adding 5 ml of 5 M NaOH solution. The mixture was incubated at 37 °C for different periods of time. After incubation, the mixture was heated in an oven at 75 °C and crushed. Crushed mixture (20 g) and 100 ml acetonitrile were added to extract any remaining ZEN and blended in an incubating shaker (ZHCHENG Inc., Shanghai, China) for 20 min at room temperature. The extract was filtered through glass microfiber filters (934-AH, Whatman). This filtrate (10 ml) was diluted with 40 ml 0.01 M PBST solution (8 g NaCl, 0.2 g KCl, 0.2 g KH_2_PO_4_, 1.16 g Na_2_HPO_4_·12H_2_O and 2 ml Tween-20 dissolved in water to total volume of 1 l). The dilution (25 ml) was washed using a Zearalenone Immunoaffinity Column (Huaan Magnech Bio-Tech, China) according to the manufacturer’s recommendations, and the ZEN concentration was quantified using the HPLC system (“High-performance liquid chromatography (HPLC) analysis” section). ZEN concentration (μg/kg) = remaining ZEN concentration (μg/ml) demonstrated by HPLC × 1000.

## Results

### Construction of expression plasmids

**Fig. 1:**
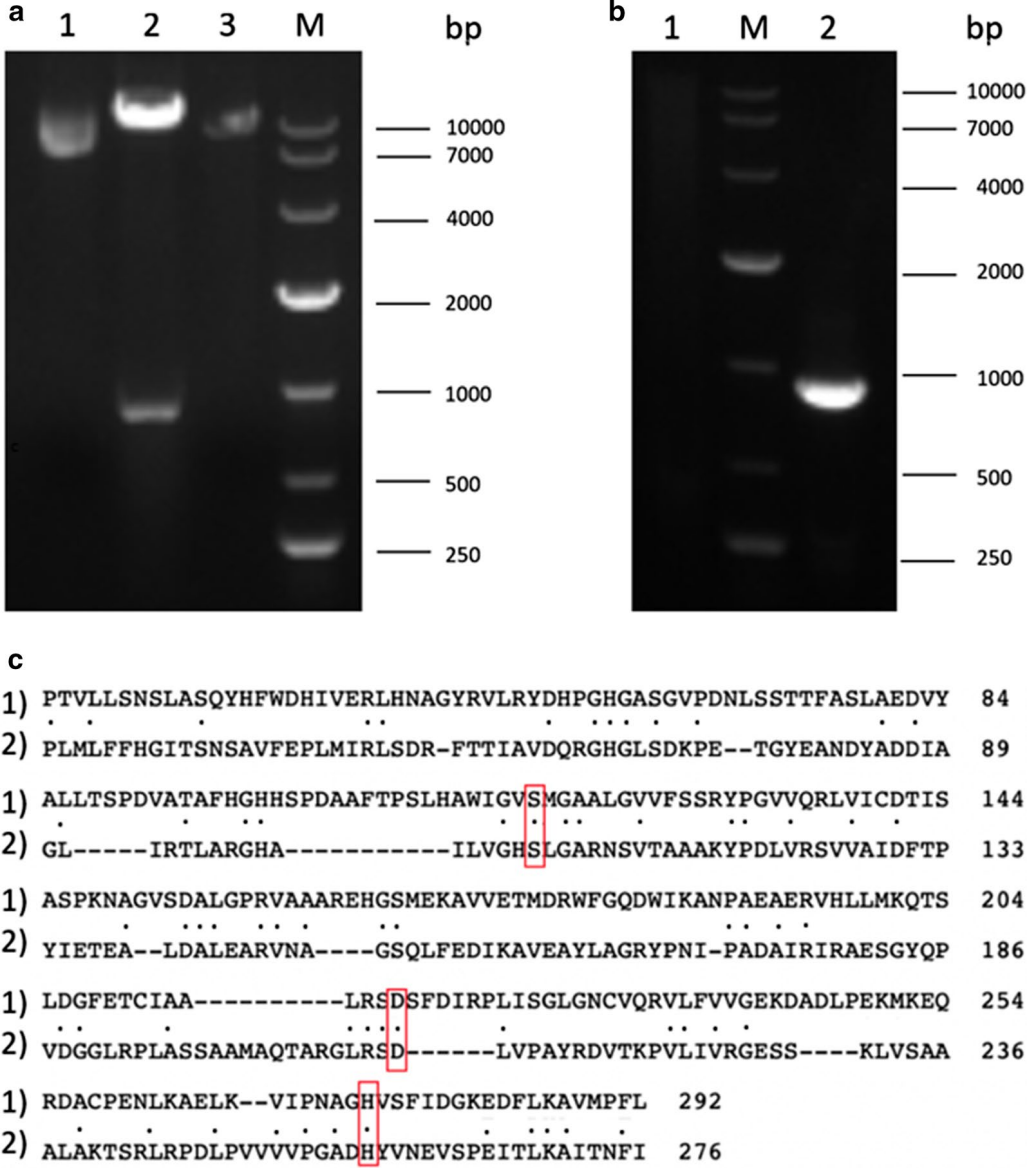
**a** Electrophoresis analysis of expression plasmid. lane M: marker; lane 1: recombined plasmid; lane 2: recombined plasmid was digested by *Eco*RI and *Not*I; lane 3: pPIC9K was digested by *Eco*RI and *Not*I. **b** Electrophoresis analysis of PCR. lane M: marker; lane 1: PCR of *P. pastoris*; lane 2: PCR of recombinant *P. pastoris*. **c** Alignment of the deduced amino acid sequence of ZENC (1, upper line) with 2-(acetamidomethylene) succinate hydrolase from *Mesorhizobium japonicum* (2, lower line). Identical (.) are shown. Residues that comprise the catalytic triad of a serine hydrolase family are boxed

Total of 196 codons of ZENC were optimized according to the codon usage of *P. pastoris*. After optimization we reduced the GC content from 57.71 to 44.81% and increased the codon adaptation index from 0.58 to 0.95. The gene fragment was synthesized and successfully cloned into pPIC9K, fused in-frame with the α-factor signal peptide. Based on amino acid analysis, the deduced mature ZENC protein contained 296 residues. The deduced amino acid sequence of ZENC was analyzed by using BLAST server (https://www.ncbi.nlm.nih.gov/BLAST) and shows its highest identity (97%) with a hypothetical protein from *N. tetrasperma*, and identity of 29% with an experimentally-verified zearalenone hydrolase (ZHD101) from *Clonostachys rosea*. The result of amino acid sequence analysis also revealed that ZENC have a significant level of similarity (23% of identity) to a 2-(acetamidomethylene) succinate hydrolase (Accession Number: WP_010914326.1) from *Mesorhizobium japonicum.* The conserved domain search showed that these two enzymes have a common structural feature named α/β hydrolase fold. Serine hydrolase family was characterized by having a catalytic triad. The sequence alignment demonstrated that ZENC has a catalytic triad consisting of Ser^117^, Asp^141^ and His^274^ (Fig. 1c, boxed residues), so we proposed that ZENC belong to the serine hydrolase family.

### Expression and purification of ZENC

The target protein ZENC was successfully expressed and secreted by *P. pastoris*. The supernatants of transformant cultures of recombinant *P. pastoris* and of *P. pastoris* were analyzed by SDS-PAGE (not shown). The enzyme activity of the transformant cultures was 40.0 U/ml after methanol induction for 72 h in shaken flasks. ZENC from the supernatant was purified and the specific activity of the purified enzyme was 530.4 U/mg. The molecular mass of the purified enzyme was found to be about 29 kDa by SDS-PAGE.

### Characterization of recombinant protein

Degradation activity assay of ZEN showed that 99.75% of the ZEN (20 μg/ml) was degraded by ZENC in 15 min. For purifying protein, the recombinant strain was cultured in shaking flasks and induced with methanol for 3 days. Then the supernatant was precipitated by adding ammonium sulfate, desalinated by ultrafiltration, and purified by HiTrap Q HP column chromatography. The purified recombinant protein was analyzed by SDS-PAGE.

**Fig. 2:**
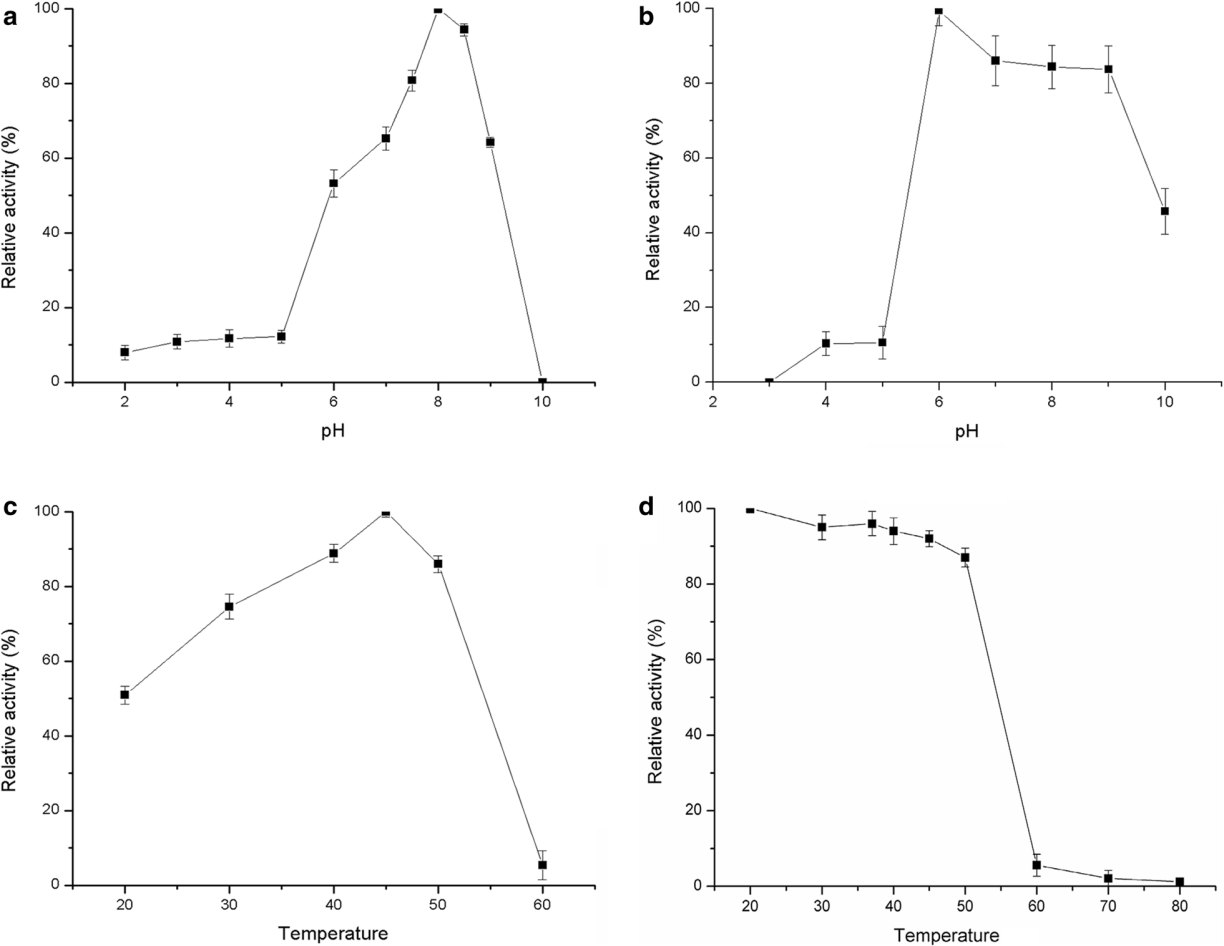
Effect of temperature and pH on the enzyme activity of recombinant ZENC. **a** Effect of pH on the enzyme activity. **b** pH stability of recombinant enzyme. **c** Effect of temperature on the enzyme activity. **d** Thermostability of recombinant enzyme. Error bars represent the standard devision of values and every case was carried out three experiments

Recombinant protein was tested with ZEN at various temperature and pH. The optimal temperature and pH for enzyme activity were 45 °C and 8.0, respectively (Fig. 2). The thermostability of ZENC was good at the temperature ranging from 20 to 50 °C and the activity of enzyme was almost lost at 60 °C for 1 min. ZENC was stable at pH range from 6.0 to 9.0, and the highest pH stability was achieved at pH 6.0. The stability of ZENC at 37 °C was shown in Table 1. The kinetic parameters of ZENC with ZEN were determined at pH 8.0 and 45 °C. K_m_ was 38.63 ± 5.868 μM, and V_max_ was 23.8 μM/s/mg. The effect of different metal ions or chemical reagents on the activity of purified ZENC was determined at a final concentration of 5 mM (Table 2). Mg^2+^, Na^+^ and Ca^2+^ had no effects on the enzyme activity. Mn^2+^ inhibited the enzyme activity partially. The enzyme activity was almost completely lost with the presence of Cu^2+^, SDS and EDTA. A decline trend of enzyme activity was observed as the concentration of Zn^2+^ increased from 5 to 15 mM.

**Table 1:** Stability of ZENC at 37 °C

Time (h)	Relative activity (%)*
0	100
6	79.5 ± 2.69
12	66.7 ± 3.01
24	40.3 ± 2.24
48	9.1 ± 1.38

* Values represent mean ± SD (n = 3) relative to 0 h sample

**Table 2:** Effect of ions and chemicals on ZENC

Chemicals	Concentration (mM)	Relative activity (%)*
Zn^2+^	5	77.3 ± 2.51
Zn^2+^	10	36.4 ± 2.64
Zn^2+^	15	17.8 ± 2.96
Cu^2+^	5	1.6 ± 0.90
Mn^2+^	5	44.9 ± 1.08
Mg^2+^	5	103.2 ± 1.24
Na^+^	5	99.6 ± 0.88
Ca^2+^	5	94.8 ± 1.37
SDS	5	3.1 ± 1.16
EDTA	5	9.211 ± 2.84

* Values represent mean ± SD (n = 3) relative to untreated control samples

### Expression of ZENC in a 30-l fermenter

**Fig. 3:**
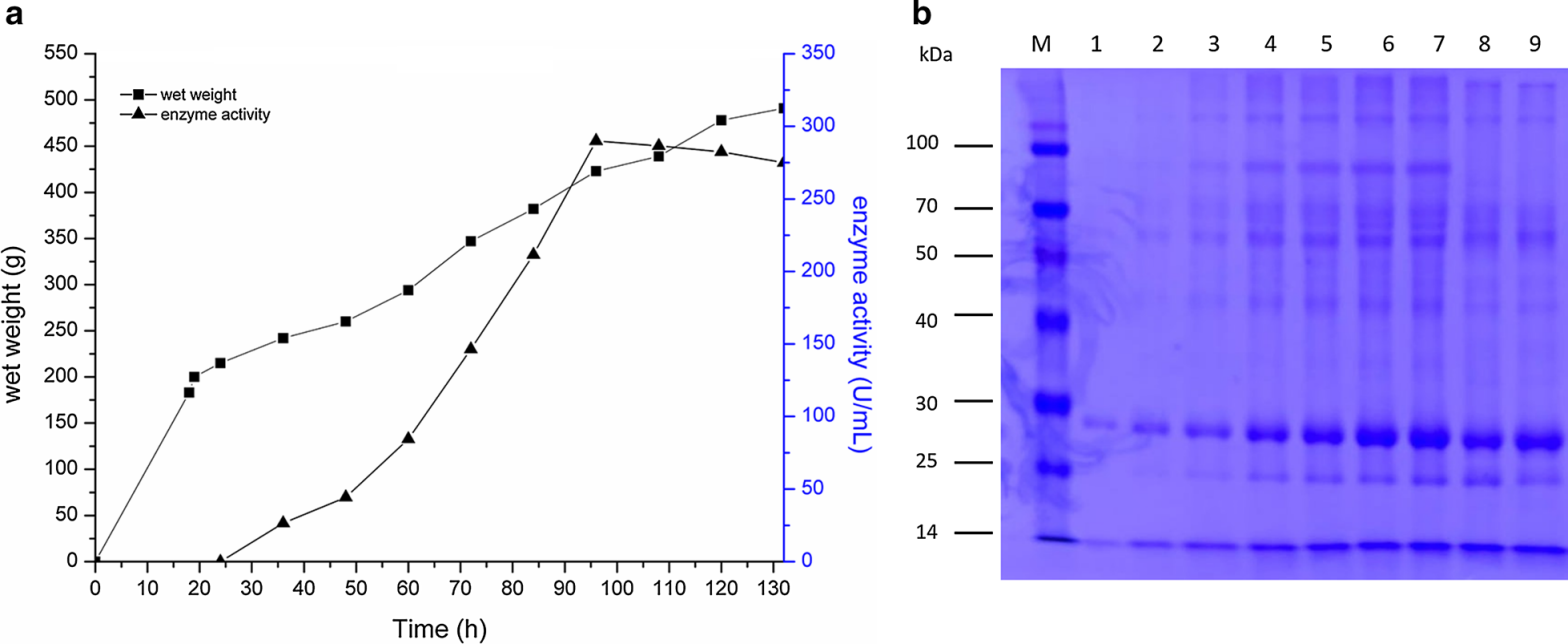
High-density fermentation of recombinant *P. pastoris* expressing ZENC in a 30-l fermenter. **a** Wet weight of cells and enzyme activity. **b** SDS-PAGE analysis of culture supernatant from the 30-l fermenter at different periods of incubation time; Lanes 1–9, sample supernatants from culture induced with methanol for 12, 24, 36, 48, 60, 72, 84, 96 and 108 h, respectively

A high-density cell fermentation of *P. pastoris* was carried out in a 30-l fermenter for high expression of ZENC. The methanol induction time was up to 100 h and fermentation broth collected every 12 h were analyzed (Fig. 3). The target protein was not detected before methanol was fed into the fermenter. Enzyme activity was analyzed by HPLC. The activity of ZENC reached its highest level after 72 h of induction: the wet weight of cells reached 485 g/l and the maximum enzyme activity was 290.6 U/ml. The enzyme activity in the fermenter was 7.27-fold that in shaken flask fermentation (40.0 U/ml).

### Application of ZENC on degradation of ZEN in DDGS, maize by-products and corn bran

**Fig. 4:**
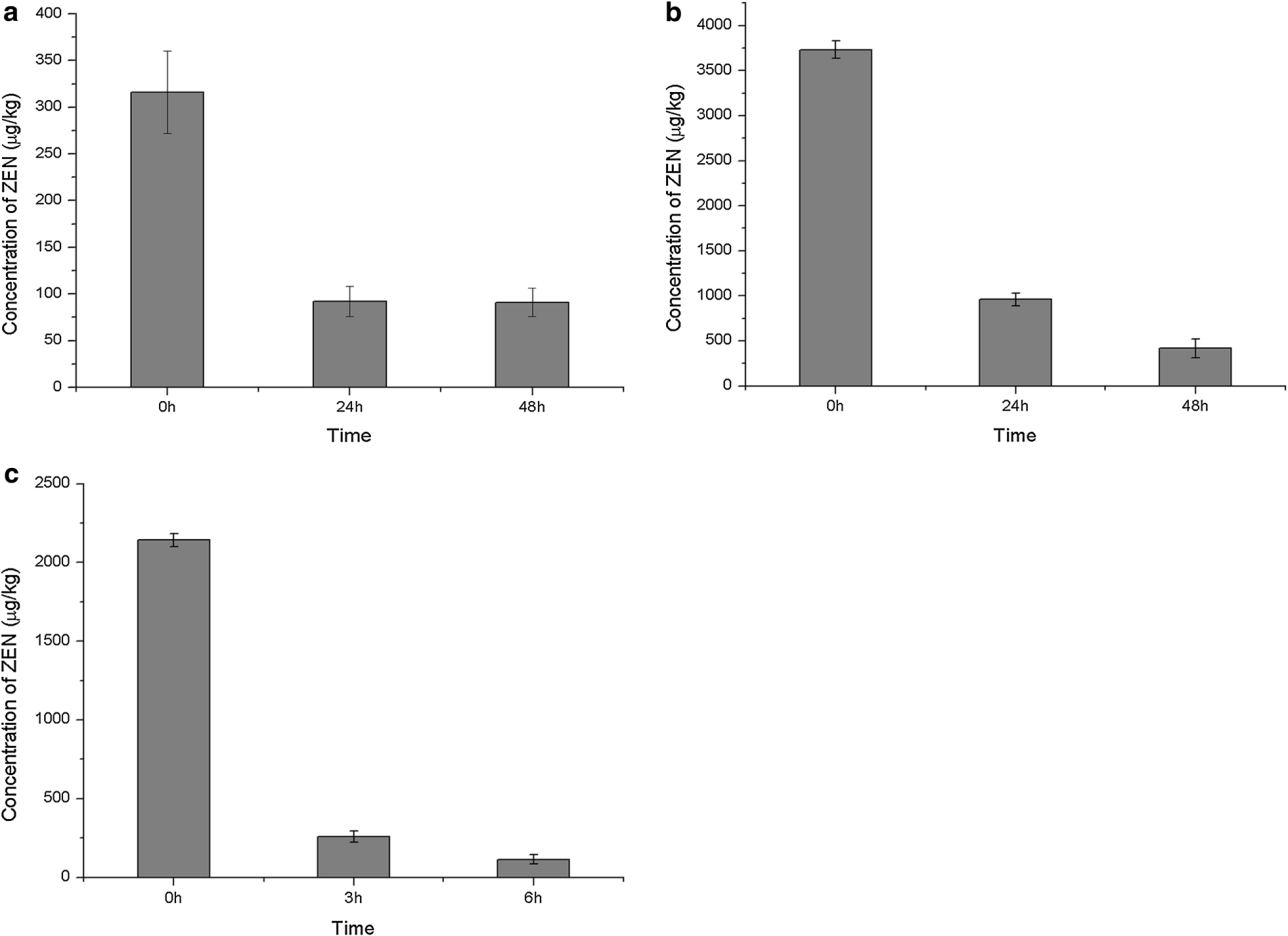
Zearalenone (ZEN) degradation in different substrates on addition of ZENC. **a** ZEN degradation in DDGS. **b** ZEN degradation in maize by-products. **c** ZEN degradation in corn bran

We added recombinant ZENC to ZEN-containing DDGS, maize by-products and corn bran. All samples were processed at 37 °C, pH 8.0. The DDGS sample containing ZEN about 316 μg/kg was treated for 24 h, and the residual ZEN concentration was reduced to the level of 92 μg/kg. The ZEN concentration did not decrease further even after the degradation was performed for 48 h. Maize by-products containing ZEN of 3732 μg/kg were treated under the same conditions and the remaining concentration of ZEN was decreased to 416 μg/kg after degradation for 48 h; the level of degradation was thus 88.9%. For corn bran, 88.0% of the ZEN (initial concentration 2142 μg/kg) was removed after 3 h of degradation, and the ZEN concentration was down to 113 μg/kg after 6 h of degradation. The degradation level of ZEN in corn bran was 94.7% (Fig. 4).

## Discussion

Methods of degrading ZEN in cereal have attracted increasing attention in the past two decades (Higa et al. 2003; McCormick 2013). Currently, most biodegradation methods of ZEN applied in practice involve adding a microbial strain that can metabolize ZEN to a nontoxic compound (Fu et al. 2016). Cell-free biodegradation systems have seldom been used to deal with ZEN contamination. Extensive work is required to screen for strains having a particular function, and genetically modified organisms are strictly regulated. All these factors increase the practical cost, so enzymatic degradation of ZEN is considered as a promising method to solve problems of ZEN contamination.

Expression of proteins in eukaryotic expression systems is widely used. *P. pastoris* shows high level protein expression which can be induced by methanol (Love et al. 2017). Its protein processing system was suitable for protein expression such as signal peptide cleavage, protein folding, post-translational modifications inside the cell and the ability of secreting protein into the medium with normal function (Jia et al. 2013; Liu et al. 2013). We employed *P. pastoris* as a secretory expression system to express the ZEN degrading enzyme *N. crassa* ZENC. From SDS-PAGE (Fig. 3), it can be clearly seen that ZENC was expressed at a high level in the supernatant of *P. pastoris* cultures and this would be beneficial for separating the target protein.

The activity of the recombinant protein reached 40.0 U/ml in shaken flask fermentation. Determined by HPLC, 82% of 10 μg ZEN was degraded by ZENC within 10 min and 99% of 10 μg ZEN was degraded within 15 min, while it took 30 min for ZHD to degrade the same amount of ZEN (Xiang et al. 2016). To our knowledge, the activity of the recombinant ZEN degrading enzyme reported here is higher than those of all other ZEN degrading enzymes reported previously. We determined the K_m_ and V_max_ values of this recombinant enzyme toward ZEN at pH 8.0, which were 38.63 ± 5.868 μM and 23.8 μM/s/mg, respectively (enzymes with low K_m_ having have high affinity for the substrate) (Johnson 2013; Kang et al. 2008; Seibert and Tracy 2014). The optimal temperature and pH of ZENC activity were 45 °C and 8.0 respectively. A Zearalenone lactonohydrolase ZHD101 from *Clonostachys rosea* has a lower K_m_ (8.5 μM) toward ZEN at pH 8.5 and the optimal pH of ZHD101 expressed in *Escherichia coli* was 10.5 (Takahashi-Ando et al. 2004). The enzyme activity of ZENC (relative to maximum activity) was over 85% at 40–50 °C, while ZHD101 had maximal activity at 37 to 45 °C, so ZENC is more thermostable. The enzyme activity of ZENC was sharply and irreversibly reduced at pH values below 5 or at temperature of more than 60 °C (Table 3). Various research indicates that the ratio of positively and negatively charged residues on the surface of a protein may be related to its pH adaptation (Mamo et al. 2009), and that the surface amino acids of a protein are correlated with its adaption to extreme temperatures (Feller 2003; Sterner and Liebl 2001). The predicted pI of ZENC was 5.71 determined by ExPASy (https://web.expasy.org/protparam/) and the enzyme activity of ZENC was significantly decreased below pH 6.0. We suggest that a negatively charged surface of the protein has an advantageous effect on the enzyme activity of ZENC.

**Table 3:** Comparison of this work with literature reports on zearalenone degrading enzymes

Enzyme name	Organism	Degrading properties	Reference
Laccase	*Streptomyces coelicolor*	Incubation at pH 4.5, 37 °C for 24 h, 100% of ZEN (6 × 10^−4^ μg/ml) was degraded	Novozymes A/S (2009)
Laccase	*Trametes versicolor*	Incubation at pH 5.2, 30 °C for 4 h, 58% of ZEN (9.36 μg/ml) was degraded	Novozymes A/S (2009)
2cys-peroxiredoxin	*Acinetobacter* sp. SM04	Incubation with H_2_O_2_ (20 mM) at pH 9.0, 30 °C for 4 h, 95% of ZEN (20 μg/ml) was degraded	Yu et al. (2012)
Lactonehydrolase	*Clonostachys rosea*	Incubation at pH 9, 37 °C for 30 min, 100% of ZEN (20 μg/ml) was degraded	Xiang et al. (2016)
		Optimal temperature was 37 to 45 °C; optimal pH was 10.5; inactivated at 50 °C or at pH 4.5	Takahashi-Ando et al. (2004)
Lactonehydrolase	*Neurospora crassa*	Incubation at pH 8.0, 45 °Cfor 15 min, 99.75% of ZEN (20 μg/ml) was degraded	
		Optimal temperature was 45 °C; optimal pH was 8.0; inactivated at 60 °C or at pH 5	This work

To our knowledge, this is first report that ZENC has been expressed in *P. pastoris* using high-density fermentation and applied to reduce the amount of ZEN from ZEN-containing materials such as maize and its by-products. We explored the effect of ZEN degradation by adding recombinant enzyme into DDGS, maize by-products and corn bran. High concentrations of ZEN are commonly reported in maize ingredients, and this limits their practical use. After the pretreatment with ZENC, the final ZEN concentrations in DDGS, maize byproducts and corn bran were 92 μg/kg, 416 μg/kg and 113 μg/kg, respectively. The maize was contaminated by the *Fusarium* existed in soil during the growing process, and the most of zearalenone was distributed on the surface of the corn. The degrading effect of corn bran was better than that of DDGS or maize by-products. We proposed that the high concentration of zearalenone and the simple ingredient contribute to the degrading effect of corn bran. The DDGS and maize by-products are products produced from maize, and the ingredients of DDGS and maize by-products are complex. These complex ingredient may coating the corn bran then inhibiting ZENC to reach the zearalenone, so the degrading performance of DDGS or maize by-products was not as good as that of corn bran. The maximum limits for ZEN in food and feeds differ in various countries. For example, the limit is 100 μg/kg for cereals and derived products in Italy, 1000 μg/kg for compound feeds in Japan, and 50 μg/kg for cereal products in France (Zinedine et al. 2007). Our results demonstrate that ZENC is a promising enzyme used as an additive in the food and animal feed industries, especially in corn processing. *P. pastoris* is suitable for expressing ZENC, which has promising pH and thermostability for industrial application.

## Abbreviations

ZEN: zearalenone
HPLC: high-performance liquid chromatography
DDGS: distillers dried grains with solubles
YPD: yeast extract peptone dextrose medium
BMGY: buffered glycerol-complex medium
